# Macrophage Migration Inhibitory Factor in Major Depressive Disorder: A Multilevel Pilot Study

**DOI:** 10.3390/ijms232415460

**Published:** 2022-12-07

**Authors:** Caroline Swoboda, Lena Deloch, Claudia von Zimmermann, Tanja Richter-Schmidinger, Bernd Lenz, Johannes Kornhuber, Christiane Mühle

**Affiliations:** 1 Department of Psychiatry and Psychotherapy, Universitätsklinikum Erlangen, Friedrich-Alexander-Universität Erlangen-Nürnberg (FAU), Schwabachanlage 6, D-91054 Erlangen, Germany; 2 Department of Addictive Behavior and Addiction Medicine, Central Institute of Mental Health (CIMH), Medical Faculty Mannheim, Heidelberg University, J 5, D-68159 Mannheim, Germany

**Keywords:** major depressive disorder, macrophage migration inhibitory factor (MIF), biomarker, inflammation

## Abstract

Macrophage migration inhibitory factor (MIF) is a controversially discussed inflammatory marker in major depressive disorder (MDD). While some studies show an association of high MIF protein levels with depression, animal models have yielded conflicting results. Thus, it remains elusive as to whether MIF plays an anti- or pro-depressive role. Therefore, we aimed to examine the potential of MIF at the genetic, expression and protein levels as a risk factor and biomarker to diagnose, monitor, or predict the course of MDD. Patients with a current major depressive episode (*n* = 66 with, and *n* = 63 without, prior medication) and remitted patients (*n* = 39) were compared with healthy controls (*n* = 61). Currently depressed patients provided a second blood sample after three weeks of therapy. Depression severity was assessed by self-evaluation and clinician rating scales. We genotyped for three *MIF* polymorphisms and analyzed peripheral *MIF* expression and serum levels. The absence of minor allele homozygous individuals in the large group of 96 female patients compared with 10–16% in female controls suggests a protective effect for MDD, which was not observed in the male group. There were no significant group differences of protein and expression levels, however, both showed predictive potential for the course of depression severity in some subgroups. While MIF protein levels, but not *MIF* expression, decreased during treatment, they were not associated with changes in depression severity. This project is the first to investigate three biological levels of MIF in depression. The data hint toward a genetic effect in women, but do not provide robust evidence for the utility of MIF as a biomarker for the diagnosis or monitoring of MDD. The observed predictive potential requires further analysis, emphasizing future attention to confounding factors such as sex and premedication.

## 1. Introduction

Major depressive disorder (MDD) is a severe disease, which affects more than 185 million people worldwide—depressive disorders in general affect around 280 million people—according to the World Health Organization [1]. Currently, MDD is a clinical diagnosis. Biomarkers are sought to allow for an objective diagnosis, endophenotyping, prognosis and therapeutic monitoring, based on the observation that MDD patients differ significantly in their biological characterization from healthy controls [2]. A heritability of MDD between 31 and 42% has also motivated the search for genetic risk and protective factors [3].

The pathomechanisms of depression are not yet sufficiently understood and are affected by disturbances of several systems including monoamines, oxidative pathways, the hypothalamic–pituitary–adrenal axis, neurotrophic homeostasis, ceramide-sphingomyelin metabolism and inflammatory processes [4,5,6,7,8,9,10,11]. In addition to current pathophysiological alterations, prenatal factors such as the androgen load in utero have been shown to modify social behavior [12] and the risk for depression and suicide in adulthood [13,14]. Strategies to promote stress-reduction during pregnancy are, therefore, attempted to diminish the negative effects of an unfavorable intrauterine environment [15]. The inflammation hypothesis is supported by studies which have reported an increased prevalence of major depressive episodes (MDE) in people with chronic inflammation as summarized in [16]. Additionally, e.g., melanoma or hepatitis C patients, who are treated with the cytokine interferon α, have a high risk of MDE as an adverse side effect [17]. This risk can by lowered by pretreatment with the antidepressant paroxetine [18]. An explanation for the promotion of MDE by cytokines could stem from the induction of the ubiquitous enzyme indoleamine 2,3-dioxygenase (IDO) by cytokines accompanied by the onset of depressive-like behavior in a mouse model. IDO catalyzes the degradation of tryptophan, the precursor of serotonin, in brain cells via the kynurenine pathway, thereby additionally causing neurotoxic metabolites. The decreased serotonin production establishes a connection of cytokines to the monoamine deficiency hypothesis of depression [19].

The macrophage migration inhibitory factor (MIF) is a protein that functions as both a cytokine and a hormone [20] and its effect can be pro- and anti-inflammatory [21]. MIF is particularly expressed by cells and tissues, which are in contact with the environment, and in organs, which participate in the stress response [22]. It is expressed constitutively and its release from intracellular storage pools of central and peripheral sources is initiated by inflammatory stimuli [22,23]. Furthermore, MIF physiologically antagonizes glucocorticoids [22]. Interestingly, low glucocorticoid concentrations, in turn, promote MIF production, which leads to a system of counter-regulation adjusting inflammatory and immune reactions [24]. In addition, MIF was found to play a role in inflammation, sepsis, and innate immunity. For diseases which affect these systems, higher MIF protein levels correlate with more severe symptoms and poorer outcome [25].

MIF is the subject of research in various neurological diseases such as multiple sclerosis [26], Alzheimer’s disease [27], and MDD [28]. Interestingly, in MDD, MIF is expressed in the brain predominantly in areas connected to behavioral symptoms; it has a link to the hypothalamic–pituitary–adrenal axis and shows an association with neurogenesis by inducing production of brain-derived neurotrophic factor [28].

In depressed patients, higher MIF baseline levels were found compared with healthy controls [29]. Higher MIF levels were also associated with higher depressive symptoms in healthy university students [30]. Consistent with these observations, MIF knock-out mice of both sexes showed decreased depressive-like behavior in the forced swim test [31]. In contrast, other studies on MIF knock-out mice (sex not reported) have observed increased depressive-like behavior [32,33]. In addition, after intracerebroventricular injection of MIF into male rats, the animals showed reduced depressive-like behavior in the forced swim test [32]. Thus, literature points towards a controversial role of MIF with pro- or anti-depressive effects [34]. However, a recent review assumed a pathogenetic role in human studies despite the disputed animal models [20].

Furthermore, there is evidence for the use of *MIF* mRNA expression to predict treatment response in depressed patients. Cattaneo et al. showed, in 2013, that non-responders to pharmacological therapy had higher relative baseline values of interleukin-1β (*IL-1*β), tumor necrosis factor-*α* and *MIF* mRNA in peripheral blood cells. *IL-1*β and *MIF* mRNA levels decreased during treatment but without relation to response [35]. In 2016, Cattaneo et al. published absolute cut-off values for mRNA levels of *IL-1*β and *MIF* to predict the responder probability to antidepressant treatment [36].

Finally, there is an association of MIF with depression at the genetic level: women with type 2 diabetes mellitus carrying the rs755622 C allele within the *MIF* gene presented a higher risk of depression [37].

Taken together, there are heterogeneous data with respect to MIF’s role in depression. It is, so far, not clear whether mimicking or inhibiting MIF, for example, via small molecules or anti-MIF nanobodies [38], would be beneficial for patients. The inconsistency of reports points towards confounding factors and the need for further investigation in larger cohorts, taking into account, for example, sex and pre-medication. The inclusion of remitted patients allows for a differentiation between state versus trait markers. Moreover, to our knowledge, there are no studies of MDD, which examined MIF at three different biological levels—*MIF* genetics, *MIF* expression and the MIF protein—and their interaction. In a highly exploratory approach, we, thus, aimed to characterize MIF in our cohort of depressed patients, remitted patients and controls. We explored the potential of MIF polymorphism as genetic risk or protective alleles for depression, as well as the utility of peripheral transcriptomic and proteomic MIF levels as a biomarker for MDD and predictor of treatment response, to guide selection for the personalized treatment for patients with MDD.

## 2. Results

### 2.1. Cohort Characteristics

The study included a total of 230 patients and controls (Table 1). There was no group difference for age and total educational years. However, premedicated patients presented with a higher body mass index (BMI) compared with the other groups, possibly due to the side effects of antidepressants. Patients with a current MDE classified with moderate to high depression severity as reflected by the scores of the three assessed scales for depression severity, Hamilton Depression Rating Scale (HAM-D), Montgomery and Åsberg Depression Rating Scale (MADRS), and Beck Depression Inventory II (BDI-II). These scores decreased from inclusion to follow-up around 3 weeks later, demonstrating the effectiveness of therapy.

When analyzing for sex differences, *MIF* expression levels were significantly higher (23% at inclusion, 24% at follow-up) in women, whereas MIF protein levels were significantly lower (18% at inclusion) in women compared with men.

*MIF* expression levels were negatively associated with BMI exclusively in healthy controls, specifically in men (total group: *rho* = −0.380, *p* = 0.003; women: *rho* = −0.233, *p* = 0.223; men *rho* = −0.413, *p* = 0.023). In contrast, MIF protein levels were positively associated with BMI exclusively in remitted patients, specifically in women (total group: *rho* = 0.530, *p* = 0.0005; women: *rho* = 0.626, *p* = 0.0004; men *rho* = 0.141, *p* = 0.679). Moreover, MIF protein levels were also negatively associated with age exclusively in healthy controls, specifically in men (total group: *rho* = −0.279, *p* = 0.029; women: *rho* = −0.079, *p* = 0.671; men *rho* = −0.459, *p* = 0.011).

### 2.2. Influence of MIF Polymorphisms on Risk for and Severity of Depression

MIF is encoded by the *MIF* gene (22q11.2) with three frequently analyzed single nucleotide polymorphisms (SNPs): rs755622 (within the promoter region), and two intronic SNPs rs2096525, and rs2070766. We first checked the quality of our data. No genotype frequency in the total, male, or female groups of combined cases and controls deviated significantly from the Hardy–Weinberg equilibrium. Systematic genotyping errors were, thus, unlikely. The minor allele frequencies of the healthy control subjects were similar to the CEPH collection population (CEU; European ancestry) from the HapMap Genome Browser. Despite the high linkage of the three SNPs (all *rho* > 0.8, all *p* < 0.001), the genotype distributions were not identical (Table 2).

We next compared the genotype distribution separately for men and women between patients with any lifetime MDE (either current or prior remission) and healthy controls for all three SNPs. In men, there were no significant differences between the distributions of major allele (N) carriers (NN + Nn) and individuals homozygous for the minor allele (n). However, in women, we found significantly different genotype distributions between patients with lifetime MDE and controls (rs755622: OR = 31.6, *p* = 0.0004; rs2070766: OR = 23.7, *p* = 0.002; rs2096525: OR = 40.1, *p* = 0.00006). There were no individuals homozygous for the minor allele for any of the three SNPs among the 96 female patients, whereas the number of minor allele homozygous individuals among the 31 healthy controls varied between 3 (rs2070766) and 5 (rs2096525). Despite the small total cohort size, these data suggested a protective effect of the minor allele homozygous genotype for MDD in women (Table 2).

We further analyzed the associations of the SNP rs2096525 with the highest genotype group effect with depression severity. At inclusion, male premedicated patients homozygous for the minor allele scored higher (*p* = 0.037) on the BDI-II scale compared with the other genotypes (genotype (n): median [Interquartile range]—NN (24): 28.0 [22.5–31.5]; Nn (8): 21.00 [17.5–26.5]; nn (2): 43.50 [35.0–52.0]) (*p* = 0.018 for difference between three groups; for other subgroups with respect to sex and premedication *p* > 0.29). Thus, in contrast to women, where homozygosity for the minor allele seemed protective, the only two men homozygous for the minor allele were more severely depressed. No significant genotype differences (*p* > 0.13 for subgroups) were found for the other MDD rating scales except for HAM-D scores at inclusion in two groups: In premedicated female patients, carrier status for the minor allele was associated with lower scores than major allele homozygosity (NN (25): 25.00 [21.0–28.0]; Nn (7): 21.00 [18.0–24.0]; nn (0); *p* = 0.037). Female healthy controls homozygous for the minor allele scored lower (*p* = 0.030) on the HAM-D scale compared with the other genotypes (NN (20): 1.25 [0.0–2.5]; Nn (6): 2.50 [1.0–5.0]; nn (5): 0.00 [0.0–0.0]; *p* = 0.019 for difference between the three groups). There were no significant differences between genotypes (*p* > 0.18 for all subgroups with respect to sex and premedication) in the follow-up and the absolute change of any of the MDD rating scales.

### 2.3. Association of Depression Diagnosis and Severity with MIF Protein and MIF Expression

At inclusion, MIF protein and *MIF* mRNA levels did not differ significantly between participants with and without current MDE. We first tested for combined groups followed by tests separated by sex and premedication and did not observe any significant differences (all *p* > 0.17). Thus, there were no group differences between unmedicated, premedicated, and remitted patients and controls.

Additionally, MIF protein levels at inclusion were not associated with baseline depression severity in patients and controls. At the *MIF* mRNA level, significant results were only found in male patients with current MDE: high *MIF* mRNA levels were correlated with low HAM-D scores at inclusion (*rho* = −0.333; *p* = 0.009). Subsequent differentiation by premedication showed no association in previously unmedicated patients (*rho* = −0.301; *p* = 0.135), but a significant result in the premedicated group (*rho* = −0.366; *p* = 0.033). However, the effect was not significant for MADRS and BDI-II scales.

### 2.4. Prediction of Treatment Course from MIF Protein and MIF Expression Levels at Inclusion

Analyzing the MIF protein level, we did not find any significant correlation between baseline values and depression severity at follow-up three weeks after inclusion (HAM-D, MADRS, BDI-II) in the total, sex-specific, and pre- or unmedicated cohorts (Table 3).

In the total and sex-separated groups, there were no significant correlations between baseline MIF protein values and the course of depression severity, i.e., change of depression scores between inclusion and follow up; however, we found opposite associations depending on the premedication status. In a mixed sex cohort of patients without prior medication, there was a significant positive correlation between the baseline MIF protein values and the absolute BDI-II change (*rho* = 0.286, *p* = 0.027, Table 3). High MIF serum levels at inclusion predicted a worse MDD course. The effect was also significant in the female part of this subgroup (*rho* = 0.382, *p* = 0.026), but not in men. In contrast, in mixed-sex premedicated patients, a negative correlation was observed between baseline MIF protein values and absolute changes of HAM-D (*rho* = −0.345, *p* = 0.007) and MADRS (*rho* = −0.347, *p* = 0.007) scores, i.e., high MIF serum levels at inclusion predicted a better MDD course. Separated by sex, the effect remained significant only in women for the MADRS absolute change (*rho* = −0.426, *p* = 0.024, Table 3).

Similar to the protein level, there was no correlation between baseline *MIF* gene expression and depression severity at follow-up in the sex-mixed and -separated groups. However, in patients without prior medication, baseline *MIF* mRNA values correlated positively with depression severity at follow-up assessed by the HAM-D scale (*rho* = 0.311, *p* = 0.017, Table 4). Separated by sex, the effect was only significant in women (*rho* = 0.383, *p* = 0.025).

Analyzing the predictive potential of the *MIF* expression at baseline for the change in depression severity, we found a positive correlation for the HAM-D (*rho* = 0.212, *p* = 0.021) and the MADRS (*rho* = 0.241, *p* = 0.008) absolute change in the total but not in the sex-separated cohort, i.e., high initial *MIF* gene expression levels predicted less improvement. Interestingly, this effect was only seen in patients without prior medication (HAM-D: *rho* = 0.381, *p* = 0.003, MADRS: *rho* = 0.378, *p* = 0.003), particularly in the female subgroup (HAM-D: *rho* = 0.428, *p* = 0.012, MADRS: *rho* = 0.439, *p* = 0.003, Table 4).

### 2.5. Associations of the Treatment Course with Changes of MIF Protein and MIF Expression

The MIF protein values showed strong correlation between inclusion and follow-up (*rho* = 0.793, *p* < 0.001; for all subgroups with respect to sex and premedication *rho* > 0.534 and *p* < 0.004). During the three weeks of therapy, MIF protein levels decreased significantly in the total group of patients (*p* = 0.020), but changes were not significant in the subgroups after separation by sex or premedication. For the relative change of MIF protein levels from baseline to follow-up, high initial concentrations were associated with a stronger decrease and low initial concentrations with a stronger increase in MIF protein (all: *rho* = −0.343, *p* < 0.001; men: *rho* = −0.363, *p* = 0.005; women: *rho* = −0.346, *p* = 0.006, for all subgroups with respect to sex and premedication: *rho* > |0.30| and *p* < 0.1). These relative changes of MIF protein were not associated with absolute changes in any of the three depression scales (all *p* > 0.5). There were also no significant correlations after separation by patients’ sex or premedication (all *p* > 0.1).

The *MIF* mRNA at inclusion correlated with the *MIF* mRNA values at follow-up in the sex-mixed sample (*rho* = 0.349, *p* < 0.001). Similar results were found in all subgroups with respect to sex and premedication (*p* < 0.038), except the lack of correlation in female premedicated patients (*rho* = 0.270, *p* = 0.165) and male patients without prior medication (*rho* = 0.095, *p* = 0.660). While overall, *MIF* mRNA levels did not change significantly during the three weeks of therapy (*p* = 0.660), analysis of the *MIF* mRNA relative change from baseline to follow-up revealed the same association as for protein levels: high baseline levels were associated with a relative decrease while low mRNA levels were associated with a relative increase (all: *rho* = −0.440, *p* < 0.001; men: *rho* = −0.402, *p* = 0.002; women: *rho* = −0.517, *p* < 0.001, for all subgroups with respect to sex and premedication: *rho* > |0.39| and *p* < 0.06). Similar to the protein data, we could also not find any significant association of these relative *MIF* mRNA changes with the absolute change of any of the depression scales (all *p* > 0.3). Additionally, testing for sex- or premedication-specific associations did not show significant results except for a negative correlation in female patients without premedication in the MADRS score (*rho* = −0.351, *p* = 0.045), i.e., an increase in the relative *MIF* mRNA values correlated with a decrease in the absolute MADRS score.

### 2.6. Association between MIF Parameters at Different Biological Levels

The analysis of associations of MIF parameters at different biological levels was based on the genotypes for the SNP rs2096525 with the highest genotype group effect.

We found 2.2-fold lower *MIF* mRNA levels in heterozygous compared with major allele homozygous female remitted patients at inclusion (*p* = 0.003). Of note, there are no minor allele homozygous female patients. For other subgroups with respect to sex and premedication at inclusion and at follow-up, there were no significant results (all *p* > 0.15). Moreover, *MIF* mRNA levels in female premedicated patients changed differently during the three weeks of therapy depending on the genotype (*p* = 0.038), even in opposite directions: they increased in heterozygous and decreased in major allele homozygous individuals. Other subgroups separated by sex and premedication had no significant results (all *p* > 0.64).

Similarly, we observed lower MIF protein levels in heterozygous compared with major allele homozygous individuals, again only in female patients, here in the following subgroups: at inclusion, in remitted patients (1.8-fold, *p* = 0.011) and a trend for current MDE patients without premedication (1.2-fold, *p* = 0.056), and at follow-up, in patients without premedication (1.4-fold, *p* = 0.050). No significant results were found in any other male or female subgroup at inclusion or follow-up (*p* > 0.16). There was no association of the genotype with the relative change of MIF protein during treatment in any of the subgroups (*p* > 0.29).

Finally and unexpectedly, there was no evidence for an association of *MIF* mRNA expression levels and MIF protein levels, neither in the total nor in the sex separated groups (all *p* > 0.26) nor in subgroups of patients and controls (all *p* > 0.06) both at inclusion and at follow-up. The *MIF* mRNA level at inclusion had an effect on protein levels at follow-up exclusively in the group of female patients without prior medication (*rho* = 0.464, *p* = 0.007; for all other subgroups with respect to sex and premedication: *p* > 0.06).

## 3. Discussion

### 3.1. Influence of MIF Polymorphisms on Risk for and Severity of Depression

There was a high linkage between the three examined *MIF* SNPs, which was also reported in the literature [39]. The SNPs were not only investigated in the context of MDD but also for inflammatory diseases. For example, the rs755622 C allele was found to be a risk factor for juvenile idiopathic arthritis [39,40], and rs755622 and rs2096525 are associated with Behçet’s disease, a chronic systemic inflammatory disorder [41].

In our study, we surprisingly did not find any minor allele homozygous individuals in the total group of 96 female patients for all three analyzed SNPs (rs755622, rs2096525, rs2070766), in contrast to 10–16% minor allele homozygous females in the healthy control group (OR up to 40) which leads to the assumption that the minor allele homozygous genotype may be protective for MDD. To our knowledge, there have only been two published studies investigating *MIF* SNPs in connection to depression or suicide. Hamidi et al. observed a higher fraction of MDD in 144 women but not 95 men carrying the rs755622 minor allele (nn + Nn vs. NN), but did not provide sex-separated data on the distribution of the three genotypes to compare with our results [37]. For our cohort, there were no significant group differences between major allele homozygotes and minor allele carriers for any of the SNPs. Moreover, the analyzed cohort consisted of an Iranian population with type 2 diabetes mellitus and might, thus, not be well comparable to our cohort where diabetic patients were excluded. Similar to our results, an association was also only found in women [37]. Another study investigated whether the *MIF* SNP rs755622 predisposes for completed suicide in the Japanese population, but could not find a different genotype distribution in suicide victims compared with healthy controls, also not separated by sex [42]. Thus, the effect of MIF genotype on the risk of depression and its possible sex-specificity and underlying mechanisms require further studies to potentially identify females at risk.

We also found associations of *MIF* genotypes with MDD rating scales at inclusion in female healthy controls where the minor allele homozygous genotype had the lowest HAM-D score, which is consistent with its potentially general protective role in women. In contrast, the minor allele homozygous premedicated men had the highest BDI-II score, emphasizing the need for sex-separated analysis in future studies.

New research also supports an important role of SNP-based heritability for response to antidepressants as 20 to 40% of its variance is due to frequent genetic variation [43]. Additional to further investigations of the three SNPs, the *MIF* gene also harbors a short tandem repeat CATT^5−8^, which will be interesting for future research [44].

### 3.2. Association of Depression Diagnosis and Severity with MIF Protein and MIF Expression

The assumption of MIF as a diagnostic marker was based particularly on two clinical studies which reported an association of higher MIF protein levels with depressive symptoms [29,30]. Moreover, higher *MIF* mRNA values compared with controls were found in depressed patients [35]. However, we could not replicate these findings neither at the transcriptomic nor the proteomic level.

At the protein level, we found no difference in MIF values between MDD patients and controls, neither in the combined groups nor separated by sex or patients’ premedication. Moreover, MIF protein levels were not associated with depression severity neither in patients nor in healthy subjects. Disagreement with the previously reported results might originate from several factors. Both published MIF protein studies had a smaller sample size. The study of Musil et al. investigated only 32 depressive and 20 healthy participants [29]. The trial of Edwards et al. studied only 28 participants with a BDI-II score ≥ 14, compared with 84 participants with few depressive symptoms (BDI-II < 14). A further limitation of this study was that only healthy university students were analyzed, and not a cross-section of society [30]. Similar to Edwards et al., Katsuura et al. examined healthy university students, but in contrast, they could not find an association between serum MIF levels and depressive symptoms, which were also evaluated by a self-rating scale [45]. Supporting the last paper, we could not show an association between high MIF protein and depressive symptoms even using the HAM-D and MADRS scales, rated by a clinician.

Apart from the pure association of MIF and depressive symptoms found in the literature, there are also supporting data of a relation between MIF and MDD in other contexts. MIF levels are slightly elevated in pregnancy, and pregnant women with MDD have even higher MIF levels than non-depressed pregnant women [46]. Furthermore, one week after an influenza virus vaccination of pregnant women—but not at baseline—a study measured higher MIF serum levels in those with greater depressive symptoms compared with those with less depressive symptoms at inclusion. It was assumed that depressive pregnant women have a sensitized inflammatory response [47]. In addition, in stroke patients, high MIF plasma levels predicted an elevated risk for post-stroke depression. This indicator could, thus, be useful for the prevention of MDD in stroke patients [48].

At the *MIF* mRNA level, our study found no difference between patients and healthy controls and, thus, could not replicate the higher *MIF* mRNA values in patients compared with controls from a published study [35]. In contrast, concerning depression severity, we even found an association of high *MIF* mRNA levels with low HAM-D scores at inclusion, but only in male MDD patients. The previous study [35] did not investigate an association with depression severity and did not perform sex-separated analyses. However, it has been shown that men can have different depressive symptoms [49], and our data further emphasize the need for sex-specific evaluation supported by well-established sex differences in depression [50,51].

In addition to elevated *MIF* mRNA levels in depressed patients in Cattaneo et al. from 2013 [35], associations of low levels of other cytokines with depressive symptoms—similar to our results—were also reported. For example, the transforming growth factor-β protein [29] and the mRNA of *IL-4* [35] were decreased in depressed patients compared with healthy controls. Both studies did not report sex-separated results. Thus, our results emphasize the importance of sex-separated analysis in future studies.

### 3.3. Prediction of Treatment Course from Baseline MIF Protein and MIF Expression Levels

To apply MIF protein and mRNA levels at baseline for treatment prediction, it is essential to consider the used antidepressant class. The review of Arteaga-Henríquez et al. from 2019 summarized that patients with high concentrations of inflammatory serum markers such as C-reactive peptide and IL-6 had a worse response to serotonergic medication compared with a low inflammatory state. However, if the antidepressive medication included dopaminergic, noradrenergic, glutamatergic, or anti-inflammatory agents, there was a better treatment response in high inflammatory state patients. Additionally, in “non-inflammatory” MDD patients, an anti-inflammatory add-on led to a worse response rate due to a weakened effect of the antidepressant or a delay of natural recovery. The few studies available for gene expression in leukocytes also indicated that patients with high inflammatory expression levels need more than a predominantly serotonergic medication for successful therapy [52].

In our study, a high MIF serum level at baseline predicted a worse MDD course in patients without premedication of a mixed-sex group, evaluated by BDI-II score. In contrast, the premedicated patients had an opposite correlation of MIF serum levels and HAM-D and MADRS scores: Here, high MIF serum levels at baseline predicted a better MDD course. Our study group of premedicated patients was characterized by a greater proportion of patients treated pharmacologically during the three weeks of therapy (100%), compared with the group of patients without prior medication (32%) who preferred non-pharmacological types of antidepressive therapy. In both groups, however, the portion of patients taking exclusively serotonergic drugs was too small (*n* = 6 without prior medication, *n* = 12 with prior medication) to draw conclusions and to compare with the data reviewed by Arteaga-Henríquez et al. [52]. Thus, future studies should be sufficiently powered for a separate analysis of patients taking only serotonergic medication and those on more than a predominantly serotonergic medication since several studies point towards differences in their response depending on the inflammatory state.

Studies specifically on MIF support the hypothesis of the review by Arteaga-Henríquez et al. [52] such as findings of higher MIF baseline levels in patients reaching remission compared with those without remission, both under a more than predominantly serotonergic medication. In one study, the therapy consisted of reboxetine (class of antidepressant: norepinephrine reuptake inhibitor) and celecoxib (nonsteroidal anti-inflammatory drug: COX-2 inhibitor) or placebo [29]. A recent study by Simon et al. is also compatible: In MDD patients who received only serotonergic medication, non-responders showed a trend for higher MIF at inclusion, and non-remitters had significantly higher MIF levels at inclusion. In the comparison group treated with a serotonergic plus an anti-inflammatory drug, an opposite effect was observed: a trend for high MIF levels was found in responders and no effect regarding remission. However, the small study consisted of only 43 MDD patients separated into two groups [53]. In our study, a limitation could be that the observation period of three weeks was too short compared with six weeks in the mentioned studies. Additionally, it is conceivable that longer observation periods could lead to more consistent results among the different MDD rating scales and to better results, especially in drug-naïve patients. A strength of our study was the three MDD scales which were rated by a clinician or by self-evaluation. In comparison, Musil et al. [29] in 2011 and Simon et al. [53] in 2021 used only either HAM-D or MADRS, rated by clinicians. Interestingly, a better long-term improvement (change in MADRS at 1-year follow-up) for predominantly female patients with mild to moderate depression and high versus low plasma MIF levels was also reported for non-pharmacological treatment in a mindfulness-based group therapy setting with a similar, albeit non-significant, trend after 8 weeks [54].

Regarding the *MIF* expression level, we found an association of high expression at inclusion and less improvement of HAM-D and MADRS scores in patients without premedication in the mixed-sex group and particularly in women. These results were similar to the data for protein levels in patients without premedication and BDI-II scores, indicating the predictive potential of MIF at both biological levels. These observations agreed with the only two studies analyzing *MIF* mRNA levels found in the literature: Cattaneo et al. from 2013 and 2016 found that patients without medication for the preceding two weeks, who did not respond to antidepressive therapy, had significantly higher *MIF* mRNA levels at baseline. However, contrary to our low number of pharmacologically treated patients, these participants received nortriptyline or escitalopram, and no sex-separated analysis was performed [35,36]. Similar to the protein level, there seemed to be confounding factors such as the type of medication and sex.

### 3.4. Associations of the Treatment Course with Changes of MIF Protein and MIF Expression

In addition to prediction, biomarkers would be also interesting to monitor the therapy response. However, although high MIF protein levels decreased during treatment, we could not observe any associations between the changes of MIF protein and the course of an MDD rating scale. These findings replicated the results of two prior studies: After eight weeks of psychotherapeutic intervention for a mixed group of patients with anxiety, depression, or stress and adjustment disorders (*n* = 168), a significant decrease in MIF protein was shown, but no association with the MADRS course. A correlation of baseline or endpoint MIF values with depression severity or course was not examined [55]. Another study treated depression of recurrent bipolar disorder for 2 months with sodium valproate or lamotrigine (*n* = 140). Here, MIF serum levels decreased during therapy with a larger reduction in the lamotrigine group. However, a correlation between MIF and HAM-D courses was not investigated [56]. On the contrary, Musil et al. examined MDD patients during five weeks of reboxetine and add-on celecoxib or placebo treatment and reported no significant MIF change between inclusion and study endpoint, and also no association between MIF course and HAM-D course [29]. A following study of the same group treated MDD patients for six weeks with sertraline, and additionally celecoxib or a placebo, but no coherent results about MIF change and connection to the MDD course were found [53].

At the expression level, we did not observe a significant change during therapy and no convincing associations with change in depression severity similar to the protein results. Although Cattaneo et al. found a general *MIF* mRNA decrease under antidepressive treatment, a correlation to the treatment course was also lacking similar to our finding [35].

### 3.5. Association between MIF Parameters at Different Biological Levels

We expected to find an influence of our analyzed highly linked SNPs on *MIF* expression and protein levels as the rs755622 has been reported to be a potentially functional polymorphism [39,57] the transition from G to C creates an activator-enhancing binding protein 4 transcription factor binding site [41].

However, we observed no significant associations of *MIF* genotypes on expression levels at inclusion and follow-up for both sexes, except for one female patient subgroup (without minor allele homozygous individuals): rs2096525 heterozygous remitted women had twofold lower *MIF* mRNA values compared with the major allele homozygous genotype at inclusion. Similarly, the study of Baños-Hernández et al. could not show a significant association of rs755622 and the *MIF* mRNA level without performing sex-separated analysis [58]. Unlike our result in healthy men and women, a study on 23 healthy individuals (sex not reported) for the rs755622 (completely linked in this group with rs2096525) revealed nearly twofold higher *MIF* mRNA values for minor allele homozygous individuals compared with similar levels of heterozygous and major allele homozygous individuals [41].

Compared with the expression level, there was a similar effect at protein level only in several subgroups of females: rs2096525 heterozygous patients had lower MIF protein values compared with the major allele homozygous patients. Similar to our data in males and further female subgroups, two studies could also not find an association of the rs755622 genotypes with MIF serum levels in systemic sclerosis [58] and multiple sclerosis patients [59]. While Baños-Hernández et al. did not analyze separated by sex [58], Castañeda-Moreno et al. performed sex-specific analysis without detecting an effect [59]. Further studies only considered the carrier status or selected samples of homozygous individuals for analysis. Two studies reported higher MIF serum levels in sex-balanced groups of patients with rheumatoid arthritis [60] or juvenile idiopathic arthritis [40] who are carriers of the rs755622 minor allele, but sex-separated analyses were not performed. Contrarily, no differences of the MIF plasma levels were found in community-acquired pneumonia patients between the rs755622 homozygous genotypes [61].

Finally, the relationship between mRNA and protein levels was analyzed. While MIF protein levels decreased significantly during three weeks of therapy, *MIF* expression levels did not change significantly and we found no significant correlation of protein and RNA levels except for an association of high expression at inclusion with high protein at follow-up for female MDD patients without prior medication. To our knowledge, the studies which investigated these two biological levels, did not analyses the mRNA and protein levels for associations [58,62,63]. These data suggest that peripheral blood MIF protein levels could be influenced by additional factors independent of measured *MIF* mRNA levels that uncouple expression and protein levels to some extent. There are several explanations possibly contributing to this disparity. First, MIF is ubiquitously and semi-constitutively expressed by many immune and non-immune cell types [64]. Thus, serum MIF levels originate from different cells, while only peripheral blood mononuclear cells were available for *MIF* RNA expression analysis. Secondly, unlike many other cytokines, MIF is produced and stored preformed in intracellular vesicles and released in conditions of stress, toxicity and apoptosis [22]. There might, therefore, be a time shift between the dynamics of *MIF* mRNA and secreted protein levels. Thirdly, in addition to the protein-coding splice variant 1 with three exons, two further splice variants are known for *MIF*. The commercial double fluorescently labelled probe applied in this project spans exons 2–3 and would, thus, detect both splice variant 1 and 3 but not 2. Since a previous study has reported even opposite effects of *MIF* SNPs on *MIF* expression depending on the platform used for assessment (microarray versus RNAseq) [65], it would be interesting to determine the association of MIF protein levels with single splice variant expressions. Moreover, MIF exists structurally as a homotrimer [66] and, therefore, dominant negative splice variants such as are known for acid sphingomyelinase [67] could additionally interfere with its protein activity. Finally, *MIF* antisense RNA (MIF-AS1), a long non-coding RNA, has been shown to inhibit MIF protein synthesis [68] and, thus, constitutes an additional modifying factor for the interplay of *MIF* expression and serum levels.

Summarizing the biological levels, results seem to depend on various factors including sex and specific diseases but also MIF-specific expression, secretion and regulation mechanisms. In consequence, analyzing separately by sex, patients and controls, and reporting detected splice variants, is essential in future projects.

### 3.6. Strengths and Limitations

Our study showed several factors of strength such as the size of the sex-balanced cohort, including unmedicated and premedicated patients and patients remitted from MDE; the assessment using three scales for depression severity; sex-separated analyses; monitoring of the treatment course; and, especially, the investigation of the three biological levels—genetics, transcription and protein for MIF. The emerging role of MIF as a target for sex steroids including fluctuations during the female cycle and response to estrogen and progesterone treatment in rats [69] underlines the importance of sex-separated analyses, which allowed us to reveal effects limited to the male or female groups. Limitations of the study were that we could only ascertain correlations and no cause, except for the effect of SNPs. We also performed a high number of statistical tests in this exploratory approach, increasing the possibility of reporting false positive results. Moreover, the number of participants for genetic research was relatively small. In addition, the study period of three weeks was short for antidepressive therapy, and should be extended in following research.

## 4. Materials and Methods

### 4.1. Study Description

This project was based on samples and data from the CeraBiDe (“Ceramide-associated Biomarkers in Depression”) study [8,9,70,71,72,73]. Recruitment took place between January 2014 and January 2017 in accordance with the ethical principles of the World Medical Association (sixth revision of the Declaration of Helsinki, Seoul 2008, [74]) and the International Conference on Harmonization Guidelines for Good Clinical Practice (1996), [75]. The study was approved by the Ethics Committee of the Medical Faculty of the Friedrich-Alexander-Universät Erlangen-Nürnberg (FAU, ID 148_13 B, 2013) and all participants provided written informed consent. We recruited depressed patients from in- and outpatients of the Department of Psychiatry and Psychotherapy at the Universitätsklinikum Erlangen. Further interested individuals fulfilling the inclusion criteria and healthy control subjects were attracted via emails, flyers, letters, local newspapers, and internet advertisement from the local area.

All participants underwent a multi-step screening procedure. Inclusion criteria were age 18–75 years, and BMI 18.5–35 kg/m^2^. Exclusion criteria were severe somatic (e.g., cancer, diabetes), autoimmune disorders, psychiatric morbidity (with the exception of nicotine dependence, and for patients with MDD), pregnancy, breastfeeding, and use of anti-inflammatory drugs or corticosteroids within the last seven days (see Ref. [73] for details). All participants were screened using the structured clinical interview for DSM-IV (SKID-I). In total, we included 129 patients with a current MDE (63 without any antidepressants for at least two weeks, 66 subjects taking antidepressants in a stable regime for at least two weeks), 61 healthy control subjects, and 39 patients with a remitted MDD, i.e., individuals with a first MDE at an age of less than 60 years and no depressive episode during the preceding 12 months. From the group of patients with a current MDE, 59 unmedicated and 60 medicated patients participated in a direct follow-up (21 and 19 days post inclusion (median), with an IQR of 17–28 and 15–24, respectively). All patients received treatment as usual during the observation period, i.e., psychotropic drug administration was adjusted for some individuals (see Ref. [73] for details). Whole blood, behavioral scores, and other parameters were collected at the time of recruitment and at follow-up. The study sample characteristics are provided in Table 1.

### 4.2. Psychometric Scales

For diagnosis and exclusion of psychiatric comorbidities, we used the structured clinical interview from the DSM-IV (SKID-I). Depression severity was quantified using the 17-item Hamilton Depression Rating Scale (HAM-D, [76]) and the 10-item Montgomery and Åsberg Depression Rating Scale (MADRS, [77]) assessed by a clinicians, as well as the 21-item Beck Depression Inventory II (BDI-II, [78]) for self-evaluation.

### 4.3. Blood Collection and Analysis

All blood drawings were performed in the morning after overnight fasting to minimize circadian and nutritional effects and processed within two hours after blood drawing. Whole blood was collected into sodium-EDTA vials and aliquots for stored for genomic DNA isolation at −80 °C. Blood samples for RNA extraction were drawn in PAXgene TM Blood RNA tubes (Qiagen, Hilden, Germany) and stored at −80 °C. Serum vials were centrifuged for 10 min at 2000× *g* at room temperature, and the aliquoted serum samples were stored at −80 °C for later MIF assays. Routine laboratory parameters were quantified at the Central Laboratory of the Universitätsklinikum Erlangen, Germany (DIN EN ISO 15189 accredited) from separately collected vials.

### 4.4. Genotyping MIF Single Nucleotide Polymorphisms

We investigated three intronic SNPs with a minor allele frequency (MAF) >0.1: rs755622, rs2070766, and rs2096525. Primer pairs were selected to ensure that PCR products did not contain any further known variants that would impede genotyping by high resolution melting (HRM). PCR reaction conditions were optimized and checked by agarose gel electrophoresis. To isolate genomic DNA from whole blood samples, the Gentra Puregene Blood Kit (Qiagen, Hilden, Germany) was used. All reactions were performed with 4 ng of genomic DNA in a total reaction volume of 5 µL on a Roche LightCycler 480 (Roche Diagnostics, Mannheim, Germany). HRM was applied for rs755622 (C < G) and rs2070766 (G < C) in order to investigate the allele-dependent melting of PCR products (primer pairs: rs755622-F 5′-GAA CAGG CCG ATT TCT AGC C-3′, rs755622-R 5′-CCA GCA ACC GCC GCT AAG-3′; rs2070766-F 5′-TGA GCC ACC CGC TGA GTC-3′, rs2070766-R 5′-AGT TGT TCC AGC CCA CAT TG-3′). The reaction mix was composed of 1× commercial PCR buffer (Rovalab, Teltow, Germany), 2.4 mM MgCl_2_ for rs755622 and 1.8 mM MgCl_2_ for rs2070766, 0.2 mM dNTPs, 0.2 µM forward and reverse primers each, 0.03 μL CyGreen (1:100 dilution in water, Enzo Life Sciences Inc., Farmingdale, NY, U.S.A.) and 0.075 units Taq DNA polymerase (Rovalab, Teltow, Germany). The thermal cycling conditions were 2 min of initial denaturation at 95 °C, 45 cycles of amplification (10 s denaturation at 95 °C, 12 s annealing at 59 °C, and 12 s extension at 72 °C) followed by 10 s of denaturation at 95 °C, re-annealing at 40 °C, and a melting step with slow heating at 0.02 K/s until 95 °C under high resolution fluorescence recording (25 acquisitions/K). Gene scanning software (Roche Diagnostics, Mannheim, Germany) was used for the evaluation of the melting curves. Due to the fact that the variation of both SNPs is between nucleotides with the same number of hydrogen bonds, in the first HRM only homozygous and heterozygous samples can be distinguished. Therefore, 2 µL of separately produced PCR product from a control major allele homozygous individual was added to all samples after finishing the first HRM, to differentiate the homozygous minor and major allele genotypes in a second HRM run without any amplification steps. The HRM genotyping method was confirmed by restriction fragment length polymorphism analysis (restriction enzyme Alu1 for rs755622 and Hha1 for rs2070766) on an agarose gel. Since no suitable HRM product could be generated for rs2096525, quantitative PCR (qPCR) with two hydrolysis probes was conducted by using TaqPath ProAmp Master Mix and TaqMan SNP Genotyping Assay (Applied Biosystems, Thermo Fisher Scientific, Waltham, MA, U.S.A.). The reaction process consisted of 10 s polymerase activation at 95 °C, 40 cycles of amplification (15 s denaturation at 95 °C and 1 min annealing at 60 °C) and a 30 s cooling step at 40 °C. Endpoint genotyping software (Roche Diagnostics, Mannheim, Germany) was employed for evaluation. Per SNP, half of the samples were analyzed in an independent duplicate (follow-up blood samples) and discordant results were repeated until a genotype was established.

### 4.5. Quantitative PCR for MIF Expression Analysis

RNA was isolated from PAXgene TM Blood RNA tubes according to the manufacturer’s instructions (Qiagen, Hilden, Germany). The concentration of RNA was determined photometrically using a NanoDrop ND-1000 UV-Vis spectrophotometer (Peqlab, Erlangen, Germany). Two hundred and fifty nanograms of RNA were used in a 10 µL reverse transcription reaction using the High Capacity Kit Quanta cDNA Kit (Cat# 4368814, Thermo Fisher Scientific, Waltham, MA, U.S.A.) to synthesize 10 µL cDNA (10 min at 25 °C, 120 min at 37 °C, 5 min at 85 °C, and a cooling step at 12 °C) in a thermocycler (SensoQuest, Göttingen, Germany). We used qPCR with 2.25 µL of 1:48 diluted cDNA template to quantify *MIF* mRNA levels relative to the reference genes beta-actin (B-Actin), ornithine decarboxylase 1 (ODC1) and beta-2-microglobulin (B2M), with established primers and probes (B-Actin-F 5′-GTC TTC CCC TCC ATC GTG-3′, B-Actin-R 5′-AGG TGT GGT GCC AGA TTT TC-3′, B-Actin-probe Cy5- 5′-GAG CAA GAG AGG CAT CCT CAC CCT GAA GTA-3′ -Eclipse; ODC1-F 5′-CGC TTA CAC TGT TGC TGC TG-3′, ODC1-R 5′-CAT CCT GTT CCT CTA CTT CGG G-3′, ODC1-probe HEX- 5′-TCC AGA GGC CGA CGA TCT ACT ATG TGA TGT-3′-BHQ1; B2M-F 5′-CGC TAC TCTC TCT TTC TGG C-3′, B2M-R 5′-GTC AAC TTC AAT GTC GGA TGG AT-3′, probe #42 of the Universal ProbeLibrary (Roche Applied Science, Mannheim, Germany) to quantify B2M) [79]. The qPCR reactions for analyzing MIF had a total volume of 5 µL with 2.5 µL TaqMan Fast Advanced Master Mix (Applied Biosystems) and 0.25 µL primers and probe (10 µM stock, order number Hs00236988_g1 for *MIF* spanning exons 2–3, 56 bp product, Thermo Fisher Scientific). The 7 µL reaction for the reference genes was composed of 3.5 µL TaqMan Fast Advanced Master Mix, 0.14 µL of each forward and reverse primers (10 µM stocks), 0.07 µL of each probe (10 µM). The PCR protocol for all reactions was: 2 min uracil DNA-glycosylase incubation at 50 °C, 2 min polymerase activation at 95 °C, 50 cycles of amplification (3 s denaturation at 95 °C and 20 s amplification at 60 °C) and a cooling step for 30 s at 40 °C. The reactions were conducted on a Roche LightCycler 480 (Roche Diagnostics, Mannheim, Germany) and the results were analyzed by the ‘Abs Quant/2nd Derivative Max’ mode (LightCycler Software, Roche). Samples were quantified in duplicate, all C_q_ values were corrected for efficiency determined from a dilution series, and the geometric mean of the MIF duplicates was normalized for the expression of the three reference genes (geometric mean of C_q_ values corrected for efficiency).

### 4.6. Enzyme Linked Immunosorbent Assay for MIF Serum Levels

Serum MIF levels were quantified in duplicates of 40 µL serum using the sandwich Human MIF DuoSet ELISA (detection range 0.020–20 ng/mL, intra-assay coefficient of variation (cv) 2%, inter-assay cv 3%, DY289, R&D Systems, Biotechne GmbH, Wiesbaden, Germany). All serum MIF quantifications were carried out using the same set of reagents and consumables, and performed by a single blinded operator.

### 4.7. Statistics

We analyzed the data using IBM SPSS statistics Version 21 for Windows (SPSS Inc., Chicago, IL, U.S.A.) and GraphPad Prism 7.00 (GraphPad Software Inc., San Diego, CA, USA). The genetic data are presented as absolute genotype numbers and continuous data as the median and interquartile ranges in tables as calculated by the custom tables function of SPSS. In the case of missing data points, study subjects were excluded from the specific analyses. We applied nonparametric statistical tests. Spearman’s method was employed to evaluate bivariate correlations. Group differences were tested using the Mann–Whitney U-test and Kruskal–Wallis test. Tests for deviations from Hardy–Weinberg equilibrium (Pearson’s χ^2^ test) and Armitage’s trend tests were computed using freely available online software (https://ihg.helmholtz-muenchen.de/cgi-bin/hw/hwa1.pl; Institute of Human Genetics, Helmholtz Center, Munich, Germany, accessed 29 August 2019). Differences between inclusion and follow-up were assessed by the Wilcoxon signed-rank for related samples. *p*-values less than 0.05 for two-sided tests were considered statistically significant. For transparency reasons, we did not correct *p*-values for multiple testing and, thus, reported nominal values. For the primary hypotheses, female and male subjects were analyzed together. Subsequently, explorative sex-specific analysis was performed because of the well-established sex differences in depression [50,51].

## 5. Conclusions

To our knowledge, this is the first study to comprehensively characterize MIF genetics, expression, and protein levels in a cohort of depressed patients and healthy controls including patients remitted from an MDE and explorative analyses separated by sex and pre-medication. We determined a potentially protective effect of the minor allele homozygous genotype for all three SNPs in females based on the absence of this genotype in the entire female patients’ group as well as lower depression severity of female minor allele homozygous controls. While this effect appears robust with respect to multiple testing, our observed further associations of *MIF* expression and protein levels such as their predictive potential for the course of depression severity do not withstand correction for the high number of statistical tests and, thus, require further investigation, ideally in well-characterized homogeneous subcohorts. This might also include the new subtype of masculine depression [49,80] with a possibly different underlying mechanism. We elaborately discussed the diverse spectrum of literature and our results of this highly explorative study make a further contribution towards understanding the role of MIF in MDD. Further investigations into the underlying mechanisms, e.g., of the protective genotype, could guide in the development of fast-acting and more effective antidepressants.

## Figures and Tables

**Table 1:** Cohort characteristics. (T1 at inclusion; T2 at follow-up; IQR, interquartile range; AU, arbitrary units, fold expression compared with reference genes; *p* < 0.05 bold for group and sex differences from nonparametric tests).

	Unmedicated Patients		Premedicated Patients		Remitted Patients		Healthy Controls			
Total Study Group	*n*	Median (IQR)	*n*	Median (IQR)	*n*	Median (IQR)	*n*	Median (IQR)	*p* Group	*p* Sex
Age (years)	64	47 (34–53)	66	46 (33–54)	39	49 (46–58)	61	42 (32–54)	0.098	0.085
Education (years)	57	15 (13–18)	58	14 (13–16)	35	14 (13–16)	51	15 (13–18)	0.171	**0.008**
BMI (kg/m^2^)	64	25.2 (22.5–27.6)	66	28.5 (24.4–30.4)	39	25.7 (23.0–29.1)	61	24.4 (23.0–27.7)	**0.001**	**0.003**
HAM-D T1	64	21 (19–24)	66	23 (20–26)	39	2 (0–3)	61	1 (0–2)	**<0.001**	0.728
HAM-D T2	60	18 (14–21)	60	15 (10–22)					0.189	0.072
HAM-D abs. change	60	−3.0 (−9.0–−1.0)	60	−7.5 (−11.0–−3.5)					**0.016**	0.324
MADRS T1	64	26 (23–28)	66	28 (24–34)	39	1 (0–3)	61	0 (0–2)	**<0.001**	0.890
MADRS T2	60	21 (18–25)	60	18 (13–26)					0.143	0.072
MADRS abs. change	60	−4.5 (−8.5–−2.0)	60	−8.5 (−12.0–−4.0)					**0.009**	**0.038**
BDI-II T1	64	28 (22–34)	66	29 (24–35)	39	3 (0–4)	61	1 (0–3)	**<0.001**	0.609
BDI-II T2	60	20 (15–25)	60	20 (13–31)					0.939	**0.014**
BDI-II abs. change	60	−8.5 (−11.0–−3.0)	60	−7.5 (−12.5–−2.0)					0.975	0.220
MIF mRNA (AU) T1	63	0.07 (0.05–0.10)	66	0.07 (0.05–0.10)	38	0.07 (0.05–0.12)	59	0.06 (0.03–0.10)	0.316	**0.003**
MIF mRNA (AU) T2	58	0.07 (0.06–0.12)	60	0.06 (0.04–0.12)					0.168	**0.011**
MIF mRNA rel. change	57	0.06 (−0.32–0.66)	60	−0.15 (−0.40–0.61)					0.502	0.471
MIF protein (pg/mL) T1.	63	732 (602–1145)	66	804 (586–1226)	39	762 (529–1104)	61	695 (567–919)	0.590	**0.003**
MIF protein (pg/mL) T2	60	699 (528–964)	60	699 (571–1218)					0.275	0.067
MIF protein rel. change	60	−0.07 (−0.25–0.10)	60	−0.06 (−0.19–0.14)					0.513	0.741
**Men**	*n*	Median (IQR)	*n*	Median (IQR)	*n*	Median (IQR)	*n*	Median (IQR)	*p* Group	
Age (years)	27	49 (35–53)	34	46 (33–53)	11	49 (33–53)	30	37 (30–49)	0.469	
Education (years)	23	16 (13–19)	30	14 (13–16)	10	15 (13–17)	26	17 (14–18)	0.127	
BMI (kg/m^2^)	27	25.7 (23.3–28.3)	34	28.5 (26.7–30.2)	11	25.8 (25.6–27.0)	30	25.0 (22.9–28.4)	**0.005**	
HAM-D T1	27	21 (19–23)	34	22 (20–25)	11	2 (0–3)	30	0 (0–1)	**<0.001**	
HAM-D T2	26	18 (14–20)	32	13 (9–21)					0.173	
HAM-D abs. change	26	−3.0 (−9.0–−1.0)	32	−9.0 (−11.0–−5.5)					**0.013**	
MADRS T1	27	27 (24–29)	34	27 (23–34)	11	2 (0–2)	30	0 (0–1)	**<0.001**	
MADRS T2	26	20 (18–24)	32	17 (13–23)					0.127	
MADRS abs. change	26	−5.5 (−10.0–−2.0)	32	−9.5 (−13.0–−6.0)					**0.032**	
BDI-II T1	27	28 (23–32)	34	27 (21–32)	11	1 (0–3)	30	2 (0–3)	**<0.001**	
BDI-II T2	26	18 (15–22)	32	17 (10–27)					0.402	
BDI-II abs. change	26	−9.0 (−12.0–−5.0)	32	−9.0 (−12.0–−3.5)					0.987	
MIF mRNA (AU) T1	26	0.06 (0.05–0.09)	34	0.06 (0.05–0.09)	10	0.09 (0.05–0.11)	30	0.05 (0.03–0.08)	0.224	
MIF mRNA (AU) T2	25	0.06 (0.04–0.09)	32	0.06 (0.04–0.11)					0.664	
MIF mRNA rel. change	24	0.02 (−0.34–0.69)	32	−0.14 (−0.38–0.35)					0.817	
MIF protein (pg/mL) T1.	27	900 (659–1454)	34	783 (646–1120)	11	859 (627–1425)	30	746 (653–965)	0.575	
MIF protein (pg/mL) T2	27	775 (547–1254)	32	704 (624–1165)					0.976	
MIF protein rel. change	27	−0.15 (−0.35–0.11)	32	−0.01 (−0.20–0.14)					0.301	
**Women**	*n*	Median (IQR)	*n*	Median (IQR)	*n*	Median (IQR)	*n*	Median (IQR)	*p* Group	
Age (years)	37	45 (32–53)	32	46 (32–56)	28	52 (47–63)	31	47 (32–60)	0.082	
Education (years)	34	15 (13–17)	28	14 (12–17)	25	14 (12–15)	25	14 (12–17)	0.634	
BMI (kg/m^2^)	37	24.4 (21.7–27.3)	32	27.3 (22.1–30.6)	28	25.3 (22.7–29.2)	31	24.3 (23.0–26.2)	0.161	
HAM-D T1	37	22 (19–25)	32	24 (21–27)	28	2 (1–4)	31	1 (0–3)	**<0.001**	
HAM-D T2	34	18 (14–21)	28	17 (11–22)					0.804	
HAM-D abs. change	34	−4.5 (−9.0–−1.0)	28	−4.5 (−11.5–−2.0)					0.474	
MADRS T1	37	26 (22–28)	32	28 (25–35)	28	1 (0–4)	31	1 (0–2)	**<0.001**	
MADRS T2	34	21 (18–25)	28	20 (15–29)					0.755	
MADRS abs. change	34	−4.0 (−8.0–−1.0)	28	−6.0 (−10.5–−2.0)					0.201	
BDI-II T1	37	29 (21–35)	32	33 (27–39)	28	3 (0–5)	31	1 (0–4)	**<0.001**	
BDI-II T2	34	22 (15–27)	28	24 (16–36)					0.318	
BDI-II abs. change	34	−8.0 (−11.0–−1.0)	28	−6.0 (−13.0–0.0)					0.921	
MIF mRNA (AU) T1	37	0.08 (0.06–0.10)	32	0.08 (0.05–0.14)	28	0.07 (0.05–0.12)	29	0.08 (0.06–0.10)	0.956	
MIF mRNA (AU) T2	33	0.08 (0.06–0.14)	28	0.07 (0.05–0.16)					0.347	
MIF mRNA rel. change	33	0.18 (−0.30–0.66)	28	−0.18 (−0.47–1.47)					0.543	
MIF protein (pg/mL) T1.	36	664 (455–843)	32	805 (509–1281)	28	702 (492–1067)	31	583 (480–807)	0.364	
MIF protein (pg/mL) T2	33	607 (447–823)	28	662 (532–1290)					0.230	
MIF protein rel. change	33	−0.05 (−0.23–0.09)	28	−0.10 (−0.19–0.13)					0.908	

**Table 2:** Genotype frequencies and odds ratios (OR) for NN + Nn vs. nn for *MIF* SNPs suggest a protective effect of the minor allele homozygous genotype for MDD in women.

			rs755622		rs2070766		rs2096525	
		*n*	NN/Nn/nn	MAF	NN/Nn/nn	MAF	NN/Nn/nn	MAF
Remitted & current MDE patients (*n* = 168)	female	96	63/33/0	0.172	66/30/0	0.156	65/31/0	0.161
	male	72	46/24/2	0.194	48/23/1	0.174	46/23/3	0.201
Healthy control subjects (*n* = 61)	female	31	19/8/4	0.258	20/8/3	0.226	20/6/5	0.258
	male	30	18/11/1	0.217	19/10/1	0.200	20/9/1	0.183
Armitage’s trend test (NN + Nn vs. nn)	total	229	OR = 7.4	***p*** = **0.006**	OR = 11.7	***p*** = **0.006**	OR = 6.0	***p*** = **0.006**
	female	127	OR = 31.6	***p*** < **0.001**	OR = 23.7	***p*** = **0.002**	OR = 40.1	***p*** < **0.001**
	male	102	OR = 1.2	*p* = 0.880	OR = 2.4	*p* = 0.519	OR = 0.8	*p* = 0.843

Nominal *p* < 0.05 in bold. MAF, minor allele frequency; MDE, major depressive episode; MIF, macrophage migration inhibitory factor; n, minor allele; N major allele.

**Table 3:** MIF protein level at inclusion predicts absolute change of depression severity while there is no association with the score at follow-up as assessed by rating by a clinician (HAM-D and MADRS) or self-rating (BDI-II) in patients with a current MDE.

MIF Protein Level at Inclusion			Absolute Change of Score from Inclusion to Follow-Up						Sum Score at Follow-Up					
			HAM-D		MADRS		BDI-II		HAM-D		MADRS		BDI-II	
		*n*	*rho*	*p*	*rho*	*p*	*rho*	*p*	*rho*	*p*	*rho*	*p*	*rho*	*p*
Current MDE patients	All	120	−0.088	0.337	−0.129	0.159	0.070	0.444	−0.113	0.218	−0.130	0.159	−0.074	0.421
	Female	62	−0.106	0.414	−0.111	0.391	0.123	0.341	−0.067	0.605	−0.084	0.516	0.026	0.842
	Male	58	−0.006	0.966	−0.066	0.622	0.041	0.760	−0.096	0.473	−0.127	0.343	−0.084	0.533
Current MDE patients without premedication	All	60	0.204	0.118	0.122	0.353	0.286	**0.027**	0.034	0.795	0.018	0.893	0.088	0.503
	Female	34	0.194	0.272	0.226	0.199	0.382	**0.026**	0.030	0.866	0.100	0.575	0.231	0.188
	Male	26	0.135	0.510	0.101	0.622	0.099	0.330	0.090	0.663	−0.069	0.737	0.029	0.889
Current MDE patients with premedication	All	60	−0.345	**0.007**	−0.347	**0.007**	−0.139	0.289	−0.192	0.143	−0.249	0.055	−0.204	0.119
	Female	28	−0.354	0.065	−0.426	**0.024**	−0.118	0.550	−0.185	0.345	−0.287	0.138	−0.265	0.174
	Male	32	−0.236	0.194	−0.304	0.090	−0.175	0.338	−0.198	0.278	−0.245	0.176	−0.154	0.401

Rho and *p* from Spearman correlations, nominal *p* < 0.05 in bold. MIF, macrophage migration inhibitory factor; MDE, major depressive episode.

**Table 4:** *MIF* expression level at inclusion predicts absolute change of depression severity and score at follow-up as assessed by rating by a clinician (HAM-D and MADRS) or self-rating (BDI-II) in patients with a current MDE.

*MIF* mRNA Level at Inclusion			Absolute Change of Score from Inclusion to Follow-Up						Sum Score at Follow-Up					
			HAM-D		MADRS		BDI-II		HAM-D		MADRS		BDI-II	
		*n*	*rho*	*p*	*rho*	*p*	*rho*	*p*	*rho*	*p*	*rho*	*p*	*rho*	*p*
Current MDE patients	All	119	0.212	**0.021**	0.241	**0.008**	0.028	0.765	0.090	0.328	0.093	0.315	0.029	0.758
	Female	62	0.211	0.100	0.234	0.067	0.003	0.979	0.192	0.135	0.191	0.137	0.065	0.617
	Male	57	0.201	0.134	0.178	0.186	0.027	0.841	−0.049	0.758	−0.056	0.676	−0.072	0.595
Current MDE patients without premedication	All	59	0.381	**0.003**	0.378	**0.003**	0.166	0.208	0.311	**0.017**	0.248	0.058	0.213	0.105
	Female	34	0.428	**0.012**	0.439	**0.003**	0.171	0.335	0.383	**0.025**	0.284	0.104	0.173	0.328
	Male	25	0.309	0.132	0.176	0.401	0.130	0.536	0.168	0.423	0.166	0.428	0.279	0.177
Current MDE patients with premedication	All	60	−0.073	0.580	0.127	0.335	−0.072	0.586	−0.055	0.675	−0.042	0.751	−0.105	0.423
	Female	28	−0.057	0.772	−0.060	0.760	−0.213	0.277	−0.011	0.956	0.041	0.835	−0.069	0.728
	Male	32	0.162	0.377	0.183	0.315	<0.001	0.998	−0.146	0.426	−0.150	0.413	−0.204	0.262

*Rho* and *p* from Spearman correlations, nominal *p* < 0.05 in bold. MIF, macrophage migration inhibitory factor; MDE, major depressive episode.

## Data Availability

Data are available upon request.
